# IFN-gamma in the tumor microenvironment: dual roles in cancer progression and therapy

**DOI:** 10.17179/excli2025-8617

**Published:** 2025-10-14

**Authors:** Jiahui Cui, Yi Zhang, Li Yang

**Affiliations:** 1 Biotherapy Center and Cancer Center, The First Affiliated Hospital of Zhengzhou University, Zhengzhou, Henan, China; 2 Zhongyuan Cell Therapy and Immunotherapy Laboratory, Henan Academy of Innovations in Medical Science, Zhengzhou, Henan, China; 3 School of Life Sciences, Zhengzhou University, Zhengzhou, Henan, China; 4 State Key Laboratory of Metabolic Dysregulation & Prevention and Treatment of Esophageal Cancer, Tianjian Laboratory of Advanced Biomedical Sciences, Academy of Medical Sciences, Zhengzhou University, Zhengzhou, Henan, China; 5 School of Public Health, Zhengzhou University, Zhengzhou, Henan, China

**Keywords:** IFN-gamma, tumor regression, tumor progression, immunoregulation, immunotherapy

## Abstract

Interferon-gamma (IFN-γ), as a pleiotropic cytokine, plays a pivotal role in antitumor immunity. Its remarkable immunostimulatory, antiproliferative, and pro-apoptotic effects make it a promising candidate for tumor immunotherapy. Here, we highlight the dual role of IFN-γ in the tumor microenvironment during tumor development and treatment. IFN-γ can enhance antigen presentation, boost cytotoxic T cell and natural killer cell activity, and inhibit angiogenesis, promoting tumor regression and correlating with favorable therapeutic outcomes. However, prolonged exposure may induce the upregulation of immune checkpoint molecules such as programmed death-ligand 1, trigger T cell exhaustion, and recruit regulatory T cells, phenomena associated with the development of treatment resistance in cancer therapy. This dual nature poses significant challenges for harnessing IFN-γ in tumor treatment, necessitating an in-depth understanding of its mechanisms within specific microenvironments. Although numerous studies have explored IFN-γ-based tumor therapies, their outcomes have been inconsistent. Thus, although IFN-γ-based therapeutic strategies hold considerable promise, their clinical translation requires precise modulation to fully exploit its antitumor effects while mitigating potential protumor risks.

See also the graphical abstract(Fig. 1).

## Introduction

Interferon-gamma (IFN-γ), encoded by the IFNG gene, is a dimeric protein composed of two antiparallel polypeptide chains (Zaidi and Merlino, 2011[133]). It exerts pleiotropic immunomodulatory effects spanning both innate and adaptive immune responses (Ding et al., 2022[27]). Studies have shown that IFN-γ can directly trigger tumor cell senescence (Ahmetlic et al., 2021[2]) and enhance antitumor immunity (Andrews et al., 2024[5]). CD4+ T cell-derived IFN-γ can reprogram tumor-associated macrophages (TAMs) to eliminate MHC-I-deficient tumors (Kruse et al., 2023[58]). Notably, the effectiveness of immune checkpoint blockade (ICB) therapy is partially determined by IFN-γ signaling pathway, as ICB-resistant tumors frequently harbor mutations in this pathway (Shen et al., 2022[103]). Immunologically, IFN-γ extends to both tumor cells (through MHC-I upregulation) and immune cells (via macrophage activation and dendritic cell cross-presentation), working synergistically to promote CD8+ T cell antigen recognition (Garris et al., 2018[34]). However, recent evidence reveals time-dependent effects: prolonged IFN-γ signaling may activate protumor immune programs (Qiu et al., 2023[92]). Clinically, although IFN-γ-based therapeutic regimens have been partially implemented for some time, their efficacy demonstrates significant interindividual variability (Schmeler et al., 2009[99], Zibelman et al., 2023[139]). 

A complete interpretation of the principles of molecular mechanisms underlying IFN-γ-dependent antitumor and protumor effects, as well as its dual roles in patients with cancer, is critical for optimizing IFN-γ-based immunotherapy strategies. The focus of future research should lie in dissecting the dynamic regulatory mechanisms of IFN-γ during tumor evolution and clarifying its precise therapeutic targets, thereby providing a theoretical foundation for clinical translation.

This unique "double-edged sword" characteristic makes IFN-γ a focal point in cancer immunology research. This review comprehensively examines the dual roles of IFN-γ in tumor progression and therapy.

## IFN-γ Production and Regulation

**Figure 2:**
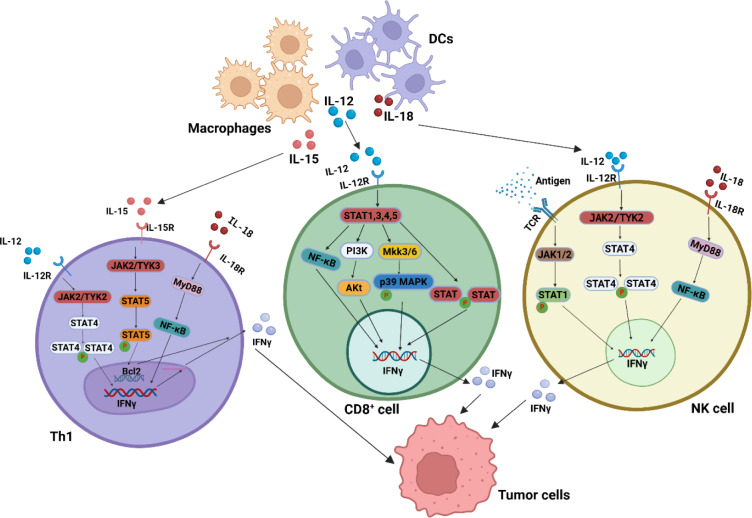
IFN-γ-producing cells and their associated signaling pathways. T helper 1 (Th1) cells, CD8+ T cells, and NK cells activate PI3K-Akt, p38 MAPK, and STAT1/3/4/5 signaling pathways upon stimulation by macrophage- and dendritic cell (DC)-derived IL-12, IL-15, and IL-18, thereby enhancing the production of IFN-γ. The released IFN-γ further exerts its effect on tumor cells.

As a critical cytokine linking innate and adaptive immune responses (Ding et al., 2022[27]), IFN-γ production in innate immunity primarily originates from natural killer (NK) cells (Sun et al., 2024[110]). In adaptive immunity, CD8+ and CD4+ T-cell subsets serve as major IFN-γ producers (Alspach et al., 2019[4], Rydyznski Moderbacher et al., 2022[96]). Other sources include certain antigen-presenting cells (APCs), such as dendritic cells, macrophages, and B cells (Burke and Young, 2019[13]). IFN-γ production must be tightly regulated through both positive and negative mechanisms in immune cells. These IFN-γ-producing cells are stimulated by interleukins (IL-12, IL-15, IL-18, and IL-21) (Kannan et al., 2011[52], Strengell et al., 2003[109]), antigens released by tumors or pathogens (Ma et al., 2023[72], Yu et al., 2024[131]), in some cases, IFN-γ itself via a positive feedback loop (Alspach et al., 2019[4]). Specific cell types and inductive signals appear to determine distinct transcription factors to initiate IFN-γ transcription. For example, IL-12 binding to its receptor on CD4+ T cells (Lu et al., 2022[69]) activates JAK2 and TYK2, leading to STAT4 phosphorylation, enhanced transcriptional activity (Thierfelder et al., 1996[112]) and subsequently, IFN-γ and IL-12Rβ2 upregulation, reinforcing IL-12 responsiveness (Afkarian et al., 2002[1]). IFN-γ promotes antigen-presenting cell (APC)-derived IL-12 secretion, creating a positive feedback loop (Garris et al., 2022[35]) (Figure 2(Fig. 2)). NKR in NK cells or TCR in T cells (Kannan et al., 2011[52]) triggers receptor signaling, activating the Src/MAPK/ERK/p38 pathway (Schoenborn and Wilson, 2007[100]). This cascade induces IFNG expression via T-bet, STAT4, AP-1, Fos, Jun, and Eomes (Jorgovanovic et al., 2020[50]).

Overall, IFN-γ production results from the synergistic effects of multiple stimulating factors. Subsequent studies are needed to comprehensively clarify the initiating signals and transcriptional regulatory mechanisms underlying its generation.

## IFN-γ Signaling Pathways

IFN-γ, a pivotal cytokine bridging innate and adaptive immunity (Ding et al., 2022[27]), exerts its biological functions through intricate signaling networks. These pathways encompass not only the canonical JAK-STAT cascade (Ealick et al., 1991[28]) but also non-canonical regulatory mechanisms (Yu et al., 2022[132]). A comprehensive dissection of IFN-γ-mediated signaling is crucial to fully understand its dual roles in tumor progression and therapeutic intervention.

### JAK-STAT pathway

IFN-γ biological activity and signaling depend on its receptor, IFNγR, which comprises two IFNγR1 and IFNγR2 chains each (Ding et al., 2022[27]). In the classical IFN-γ/IFNγR/JAK/STAT pathway, IFN-γ binds to IFNγR as an antiparallel dimer (Ealick et al., 1991[28]). JAK1 and JAK2 are respectively linked to the intracellular domains of IFNγR1 and IFNγR2. Upon ligand binding, the receptors undergo phosphorylation (Zaidi and Merlino, 2011[133]), and IFNγR2 transmits signals that promote IFNγR1-IFN-γ complex internalization. Accompanied by the translocation of the IFNγR1 extracellular domain into the intracellular compartment, JAK2 translocates from IFNγR2 to IFNγR1 owing to its stronger binding affinity for IFNγR1 (Johnson et al., 2011[49]). Activated JAK1 and JAK2 subsequently phosphorylate the intracellular domain of IFNγR1, thereby generating binding sites for STAT1 recruitment (Zaidi and Merlino, 2011[133]). Phosphorylated STAT1 translocates to the nucleus and binds to gamma-activated sequence (GAS) elements, thereby initiating target gene transcription (Sekrecka et al., 2023[101]).

IFN-γ-induced genes are collectively referred to as interferon-stimulated genes (ISGs), many of which are transcription factors that can further regulate effector gene expression (Liu et al., 2019[67]). The expression of these functionally diverse ISGs significantly influences IFN-γ stimulation outcomes (Han et al., 2023[42]).

**Figure 3:**
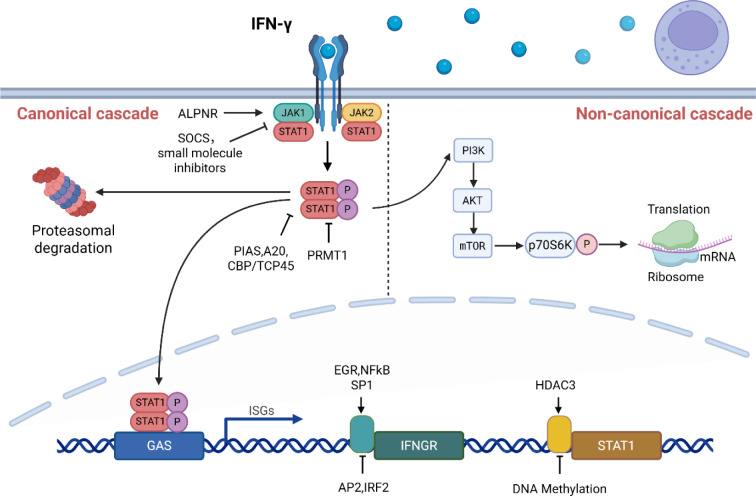
IFN-γ signaling pathways. 1. JAK-STAT pathway : (1) IFNγR: Transcriptional regulation: NFκB, EGR, and SP1 promote IFNGR mRNA transcription, while AP2 and IRF2 inhibit transcription. (2) JAK: ALPNR is essential for normal JAK function. SOCS proteins and small molecule inhibitors suppress JAK-mediated signaling. (3) STAT1: Transcriptional regulation: STAT1 promoter activity can be suppressed by methylation or activated through HDAC3-mediated deacetylation. Post-translational modifications: PIAS, A20, and CBP/TCP45 inhibit signal transduction activity by dephosphorylating p-STAT1. PRMT1 regulates STAT1 activity/stability independently of its phosphorylation state. p-STAT1 can be degraded via the proteasome pathway. 2. Non-canonical pathway : IFN-γ activates the STAT1-PI3K-Akt axis, thereby recruiting the mammalian target of rapamycin (mTOR) into interferon signaling. Additionally, the mTOR/p70S6 kinase cascade facilitates the mRNA translation of effector proteins.

The IFN-γ/IFNγR/JAK/STAT signaling pathway is stringently regulated at multiple levels. At the regulatory level of IFNGR, transcription factors NFκB, EGR, and SP1 enhance IFNGR mRNA expression (Chen et al., 2012[18]), while AP2 and IRF2 exert inhibitory effects (Chen et al., 2012[18], Wang et al., 2008[120]). Negative regulation of JAK activity is mediated by the suppressor of cytokine signaling (SOCS) proteins and small-molecule inhibitors (Liau et al., 2018[66]), whereas APLNR is essential for its normal function (Liu et al., 2022[68]). The regulatory mechanisms of STAT1 activity include: post-translational modifications mediated by PIAS (Niu et al., 2018[81]), A20 (Breitenecker et al., 2021[10]), and CBP/TCP45 (Krämer et al., 2009[57]); PRMT1-mediated regulation independent of phosphorylation status (Mowen et al., 2001[77]); and the proteasomal degradation pathway (Li et al., 2021[61]). STAT1 promoter activity can be suppressed by methylation (Xu et al., 2022[128]) or activated through HDAC3-mediated deacetylation (Yang et al., 2022[130]) (Figure 3(Fig. 3)).

In addition to the classical pathway, IFN-γ also has non-classical signal transduction pathways (Jorgovanovic et al., 2020[50]). Whether these pathways function synergistically or independently requires further investigation. In view of the significant role of IFN-γ signaling in tumor immunity, a comprehensive understanding of these regulatory mechanisms is of great importance.

### Non-canonical IFN-γ -activated pathway

IFN-γ exerts differential effects depending on exposure duration and concentration. Acute high-dose exposure induces growth arrest and apoptosis, whereas chronic low-dose exposure promotes cell survival (Cheon et al., 2023[20]). Our previous studies in the tumor microenvironment (TME) of non-small cell lung cancer revealed that IFN-γ concentration determines pathway activation: high doses trigger classical JAK/STAT signaling, whereas low doses activate ICAM1-PI3K-Akt-Notch1 cascade, increasing CD133 expression and cancer stemness (Song et al., 2019[108]). IFN-γ induces the upregulation of programmed death-ligand 1 (PD-L1) through both JAK/STAT (Han et al., 2023[42]) and PI3K-Akt pathways (Gao et al., 2018[33]). Although JAK2-mediated STAT1 phosphorylation drives gene transcription, PI3K-Akt modulates STAT1 phosphorylation levels. PI3K inhibition significantly reduces ISG expression (CXCL9/10, PD-L1), suggesting crosstalk between these pathways (Gao et al., 2018[33]). The hyperactivated PI3K/Akt/mTOR pathway (Yu et al., 2022[132]), enhances STAT1-induced ISG transcription via mTOR/p70S6K-mediated mRNA translation (Kaur et al., 2008[53]), suggesting that IFN-γ-mediated PI3K-Akt activation may amplify effector protein production (Figure 3(Fig. 3)).

## Dual Roles of IFN-γ on Tumor Progression

**Figure 4:**
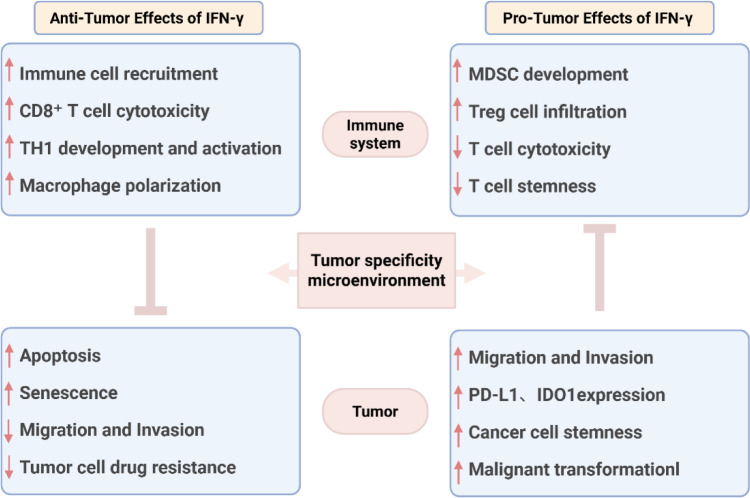
Specific mechanisms involved in the dual role of IFN-γ in exerting both anti-tumor and pro-tumor effects.

The dual effects of IFN-γ have attracted widespread attention, motivating numerous mechanistic investigations by researchers. Although many significant findings have been obtained, numerous questions remain unaddressed. Here, by discussing previously published research outcomes, we provide insights into how IFN-γ exerts its dual effects on tumors (Figure 4(Fig. 4)).

### Anti-tumor effects of IFN-γ

IFN-γ exerts antitumor effects through dual mechanisms: direct action on tumor cells and TME modulation. In tumor cells, IFN-γ suppresses proliferation by inducing apoptosis (Chaudhari et al., 2024[17]) and senescence (Homann et al., 2022[44]) while inhibiting invasion and metastasis (Chaudhari et al., 2024[17]) and reversing drug resistance (Espinosa-Carrasco et al., 2024[30]). Immunologically, IFN-γ recruits immune cells into the TME (Gocher et al., 2022[39]), promotes Th1 polarization of CD4⁺ T cells while suppressing Th2/Th17 differentiation (Gocher et al., 2022[39]) and enhances CD8⁺ T cell cytotoxicity (Shen et al., 2023[104]). Further, it drives macrophage polarization toward the M1 phenotype (Gocher et al., 2022[39]) and facilitates germinal center formation in B cells via BCL6 (Jackson et al., 2016[47]).

#### Promoting tumor cell apoptosis

Research on apoptosis is intricately linked to oncology (Moyer et al., 2025[78]). The terminology for the concept of "apoptosis" was coined over five decades ago following its discovery in both healthy tissues and malignant tumors. Apoptosis is essential not only for the maintenance of tissue homeostasis but also for tumor growth and therapy-induced tumor regression (Kerr et al., 1972[54]). The TRIM family proteins, possessing E3 ubiquitin ligase activity, play pivotal roles in diverse cellular processes including innate immunity, autophagy, intracellular signaling, and carcinogenesis (Hatakeyama, 2017[43]). In lung cancer, TRIM proteins can function as either tumor suppressors or oncogenes by modulating various signaling pathways (Zhan and Zhang, 2021[136]). Recent findings demonstrate that IFN-γ upregulates TRIM34 expression, which promotes apoptotic signaling through key regulators, thereby reducing tumor viability (Chaudhari et al., 2024[17]).

#### Activating and maintaining senescence programs in tumor cell

Cellular senescence includes oncogene activation or mitochondrial dysfunction (Gorgoulis et al., 2019[41], Lian et al., 2020[65]). Senescent cells demonstrate prominent characteristics encompass metabolic dysregulation, stable cell cycle arrest, enhanced secretory activity, elevated senescence-associated β-galactosidase (SA-β-gal) activity, and macromolecular damage (Gorgoulis et al., 2019[41], Ou et al., 2021[84]). Another defining characteristic of senescent cells is their secretion of diverse bioactive molecules, collectively termed the senescence-associated secretory phenotype (SASP) (Birch and Gil, 2020[9]). In senescent melanoma cells, cell cycle arrest is predominantly mediated by p21 (Engeland, 2022[29]). Research indicates that cytokine-induced senescence (CIS) serves as a core mechanism underlying the antitumor effects of various immunotherapies (Brenner et al., 2020[11]). Recent comparative studies between CIS (IFN-γ+TNF treatment) and therapy-induced senescence (TIS, doxorubicin/palbociclib treatment) in melanoma models revealed that all treatments induced stable growth arrest and boosted SA-β-gal activity, with all except palbociclib significantly promoting p21 upregulation. Notably, CIS-stimulated SASP factor expression and secretion levels were several-fold stronger than TIS. Experiments confirmed that conditioned media from either cytokine or palbociclib treatments could induce senescence features in melanoma cells, suggesting that IFN-γ and TNF may establish a self-sustaining senescence surveillance system to inhibit tumor progression (Homann et al., 2022[44]).

#### Inhibiting tumor invasion and metastasis

Cancer metastasis represents a critical global public health challenge, characterized by its highly heterogeneous biological nature (Traba et al., 2021[113]). Despite extensive research, developing targeted therapies against metastatic seeding and colonization remains an unresolved scientific frontier in oncology (Bergers and Fendt, 2021[7]). Consequently, elucidating the molecular mechanisms governing tumor cell dissemination and metastatic outgrowth holds profound scientific significance (Lyden et al., 2022[71]). The lymphatic system is a major route for melanoma dissemination, facilitating tumor cell spread to draining lymph nodes (Dieterich et al., 2022[26]). Lymphatic integrity, maintained through the proper organization of junctional proteins in lymphatic vessels (Dieterich et al., 2019[25]), represents a critical barrier against tumor metastasis. Recent mechanistic studies reveal that IFN-γ suppresses melanoma cell trans-lymphatic endothelial migration through a novel pathway involving AMPK signaling inhibition and subsequent upregulation of tight junction protein Claudin-3 in lymphatic endothelial cells (LECs) (Zhu et al., 2024[138]). Furthermore, emerging evidence from lung cancer research indicates that TRIM34 activation mediated by IFN-γ may effectively inhibit metastatic progression by compromising cancer cell migratory and invasive capacities (Chaudhari et al., 2024[17]).

#### Remodeling TME and overcoming ICB resistance

ICB therapy can elicit remarkable clinical responses across multiple cancer types; however, the development of resistance to this treatment approach remains prevalent (Patel and Minn, 2018[85], Shan et al., 2022[102]). In melanoma, studies have demonstrated that IFN-γ modulates immune responses following immune checkpoint inhibitor (ICI) therapy (Yamazaki et al., 2017[129]). Currently approved ICIs for patients with melanoma are as follows: (Gocher et al., 2022[39]) anti-programmed death 1 (PD-1) antibodies nivolumab and pembrolizumab, anti-PD-L1 atezolizumab (Mendoza et al., 2019[76]), anti- cytotoxic t-lymphocyte-associated protein 4 (CTLA-4) lpilimumab (Ding et al., 2022[27]), and anti-lymphocyte activation gene 3 (LAG-3) relatlimab. PD-1 inhibition elevates IFN-γ levels at tumor sites, enhancing chemokine-dependent immune cell trafficking to melanoma lesions (Peng et al., 2012[88]). Under the influence of ICIs, IFN-γ regulates inflammatory and immune responses by increasing MHC expression and antigen presentation, influencing TAMs and DCs, and promoting Th1 cell responses; however, counter-regulatory mechanisms that attenuate anti-tumor immunity are also involved (Ivashkiv, 2018[46]). Elevated IFN-γ secretion has been shown to determine effective ICI responses in melanoma through comprehensive CD8+ T cell reprogramming (Espinosa-Carrasco et al., 2024[30]). Recent studies in murine melanoma models revealed that CD8+ T cells lacking PD-1 and LAG-3 demonstrate more potent tumor clearance due to increased IFN-γ secretion (Andrews et al., 2024[5]). Furthermore, combined administration of nivolumab and relatlimab potentiates CD8+ T cell differentiation by enhancing TCR signaling and IFN-γ pathway responses, correlating with augmented T cell effector functions (Cillo et al., 2024[22]).

### Pro-tumor effects of IFN-γ

IFN-γ may paradoxically promote tumor progression under certain conditions. Notably, IFN-γ can recruit myeloid-derived suppressor cells (MDSCs) to facilitate tumor growth (Theivanthiran et al., 2020[111]). Moreover, IFN-γ stimulation enhances regulatory T cell (Treg) infiltration while impairing the cytotoxicity of T cells (CTLs) (Xie et al., 2023[126]). Moreover, it disrupts the tumor immune microenvironment by suppressing the maintenance of stem-like properties in intratumoral T cells (Mazet et al., 2023[74]). Remarkably, IFN-γ can directly exert effects on tumor cells to promote their survival and induce stemness properties. (Song et al., 2019[108]). It significantly upregulates PD-L1 (Falcinelli et al., 2023[31], Wu et al., 2024[125]) and indoleamine 2,3-dioxygenase (IDO) expressions (Schalper et al., 2017[98]) in tumor cells, and drives malignant transformation (Choi et al., 2022[21]). These changes ultimately drive immune evasion and subsequent tumor progression (Pedrosa et al., 2024[87], Wu et al., 2024[125]).

#### Promoting cancer stem cell (CSC) properties 

CSCs represent a rare population of malignant cells characterized by self-renewal capacity, pluripotency, immune privilege, high tumorigenicity, and longevity. The stem-like traits and tumorigenic potential of CSCs are partially determined by constitutive activation of highly conserved signaling pathways, including Notch, Wnt, and Hedgehog cascades (Chen et al., 2011[19]). The emerging concept of "CSC immunology" has revealed that CSCs depend on a specialized immune niche for their maintenance. In hepatocellular carcinoma (HCC), liver cancer stem cells have been identified as the primary drivers of therapy resistance, metastasis, and tumor recurrence (Nassar and Blanpain, 2016[80]). Notably, accumulating evidence demonstrates an association between the tumor-promoting effects of IFN-γ and CSC regulation. IFN-γ exposure upregulates the expression of stemness markers CD133 and CD44 in tumor cells, providing novel mechanistic insights into IFN-γ-mediated oncogenesis (Li et al., 2024[62]).

#### Upregulating PD-L1 and IDO-1 expressions

The PD-L1 signaling pathway performs a significant function in tumor immunoregulation (Majidpoor and Mortezaee, 2021[73]). The molecular mechanism of this pathway involves PD-1, which triggers downstream suppressive signals upon binding to its ligand PD-L1, leading to T cell exhaustion and functional impairment (Majidpoor and Mortezaee, 2021[73], Ping et al., 2024[89]). Within the TME, overexpression of PD-L1 in both tumor cells and antigen-presenting cells represents a critical mechanism of immune evasion. Notably, IDO-1, a key enzyme in tryptophan metabolism, effectively suppresses immune cell functions, including T lymphocytes, by depleting local tryptophan in the microenvironment (Platten et al., 2014[90]). In lung cancer animal models, IDO-1 has been demonstrated to exert dual effects of promoting immunosuppression and tumor progression (Smith et al., 2012[106]). Research indicates that IFN-γ stimulation can simultaneously upregulate both PD-L1 and IDO-1 expressions (Schalper et al., 2017[98]), indicating that this synergistic effect may constitute an important molecular basis for its tumor-promoting activity.

#### Promoting malignant phenotypic transformation and tumor progression

Intratumoral heterogeneity (ITH) significantly compromises the efficacy of anticancer therapies by mediating treatment escape mechanisms and has been established as a key driver of therapeutic failure (Vitale et al., 2021[118]). Lineage plasticity, serving as a core mechanism underlying ITH development (Bhat et al., 2024[8]), is increasingly acknowledged as a defining feature of cancer (Mehta and Stanger, 2024[75]). Although most muscle-invasive bladder cancers (MIBCs) are pathologically classified as urothelial carcinomas, they consistently exhibit significant heterogeneity at both morphological and molecular levels (Warrick et al., 2019[121]). Recent studies have stratified MIBC into six distinct transcriptional subtypes: luminal, luminal nonspecified, luminal unstable, stroma-enriched, basal/squamous (Ba/Sq), and neuroendocrine-like subtypes (Kamoun et al., 2020[51]). Notably, emerging evidence demonstrates that IFN-γ/JAK1/STAT1 signaling pathway activation drives the transition from luminal MIBC to the more aggressive Ba/Sq subtype by downregulating forkhead box A1 (FOXA1) expression in urothelial cells (Lawrence et al., 2025[59]).

Guanylate-binding protein 1 (GBP1), a GTPase, serves as a downstream effector of the IFN-γ signaling pathway (Prakash et al., 2000[91]). Current research demonstrates that IFN-γ in the breast TME upregulates GBP1 expression through activation of the IFN-γ-STAT1 signaling axis, thereby facilitating their transendothelial migration across the blood-brain barrier (BBB) (Pedrosa et al., 2024[87]). However, the IFN-γ pathway alone is insufficient to fully mediate efficient BBB penetration, as additional signaling pathways beyond IFN-γ contribute to promoting breast cancer cell migration across the BBB (Pedrosa et al., 2024[87]). Notably, the C-X-C motif chemokine ligand 9/10/11 (CXCL9/10/11)- C-X-C motif chemokine receptor 3 axis, which relies on IFN-γ activity, is highly expresse in primary breast tumors from patients with subsequent brain metastases. These findings collectively indicate that the IFN-γ signaling pathway has a substantial impact on the establishment of estrogen receptor-positive (ER+) breast cancer brain metastases (Pedrosa et al., 2024[87]).

#### Inducing immunosuppressive microenvironment formation

Excessive or sustained IFN-γ signaling activation may lead to IFN-γ-dependent pathway dysregulation, fostering the development of an immunosuppressive TME (Wawrzyniak and Hartman, 2025[122]). Within the TME, IFN-γ causes the Nod-like receptor protein 3 (NLRP3) inflammasome to be activated, accompanied by the concurrent release of heat shock protein 70 and the Wnt ligand WNT-5a (Theivanthiran et al., 2020[111]). WNT-5a further activates the Hippo/Yes-associated protein signaling pathway, triggering upregulation of C-X-C motif chemokine receptor 2 and promoting MDSC recruitment (Theivanthiran et al., 2020[111]). Furthermore, IFN-γ suppresses the maintenance and functional diversity of stem-like T cells within tumors, collectively impairing antitumor immune responses. It also downregulates the expression of NAD(P)-dependent steroid dehydrogenase-like protein (NSDHL), subsequently promoting TGF-β1 production. (Xie et al., 2023[126]). This process not only diminishes CTLs but also increases Treg infiltration, ultimately exacerbating the immunosuppressive state of the TME (Xie et al., 2023[126]).

## Dual Roles of IFN-γ in Cancer Therapy

IFN-γ is a pivotal pleiotropic cytokine that not only orchestrates the crosstalk between innate and adaptive immunity (Ding et al., 2022[27]) but also demonstrates a paradoxical dual role in cancer immunotherapy. Recently, extensive research efforts have been devoted to synergistically enhance IFN-γ-mediated antitumor efficacy while mitigating its tumor-promoting effects. These advancements have not only provided novel approaches to address clinical challenges (including therapeutic resistance and toxicity) but have also laid a solid foundation for translating IFN-γ into clinical practice to improve patient outcomes. This review systematically summarizes key mechanistic insights and research progress to provide a theoretical framework for developing more precise immunotherapeutic strategies.

### Exerting antitumor effects

Current studies have demonstrated that IFN-γ is capable of suppressing tumor development through various pathways (Chaudhari et al., 2024[17], Cheon et al., 2023[20], Homann et al., 2022[44]). Extensive recent studies aim to enhance its therapeutic effectiveness and address clinical obstacles. This review summarizes the antitumor mechanisms of IFN-γ and related research progress, these efforts are expected to facilitate clinical translation and improve prognosis for patients with cancer.

#### Combating breast cancer drug resistance and reprograming the TME

HER2-positive breast cancer represents is a particularly aggressive form of the disease (Li et al., 2024[64]). Although HER2-targeted therapy has significantly improved survival (Oh and Bang, 2020[83]), some patients fail to achieve pCR even with anti-HER2 neoadjuvant therapy (van Mackelenbergh et al., 2023[116]). Integrating HER2-targeted therapy with approaches that strengthen anti-HER2 Th1 immunity could lead to better outcomes for high-risk patients (Datta et al., 2016[23]), with IFN-γ being a key effector of Th1 immunity (Wen et al., 2025[123]). HER2 is normally degraded via the CUL5-mediated proteasomal pathway but can evade degradation by binding to Hsp90 (Pearl, 2005[86]). The Cdc37-Hsp90 complex functions as an essential molecular chaperone for HER2 stability and activity (Jia et al., 2021[48]). Recent findings show that IFN-γ disrupts the HER2-Cdc37-Hsp90 interaction, promoting CUL5-mediated HER2 breakdown and enhancing trastuzumab efficacy in resistant cells (Jia et al., 2021[48]).

Toll-like receptor (TLR) agonists have been explored as promising anticancer agents, (Rolfo et al., 2023[95]). As IFN-γ alone may be insufficient to optimally induce antitumor T cell and macrophage phenotypes and may require secondary signals from the microenvironment, combination with TLR agonists appears particularly suitable (Müller et al., 2017[79]). Research indicates that poly (γ-glutamic acid) (γ-PGA) nanoparticles (NPs) effectively trigger robust innate and adaptive immune responses (Uto et al., 2011[114]), while chitosan (Ch) activates the NLRP3 inflammasome to trigger robust IL-1β production (Bueter et al., 2011[12]). Ch/γ-PGA NPs have demonstrated synergistic effects with conventional radiotherapy in reducing systemic immunosuppression and tumor progression in 4T1 breast cancer models (Castro et al., 2020[16]), suggesting their potential as adjuvants for IFN-γ-based therapies. Recent findings in 4T1 orthotopic breast tumor models reveal the therapeutic synergy of combining Ch/γ-PGA NPs with IFN-γ. The combination therapy completely halted primary tumor growth throughout treatment and reduced pulmonary metastatic burden. Systemically, it decreased immunosuppressive myeloid cell percentages while increasing Th1 and cytotoxic T cell populations (Castro et al., 2025[15]). This combined approach represents a novel strategy for enhancing IFN-γ-based anticancer treatments.

#### Synergizing with STING agonist and ICB to enhance gastric cancer therapy 

cGAS-STING pathway activation induces multiple antitumor effects, including immune response activation, interferon production, and tumor cell death. Consequently, tumor cells often suppress this pathway to evade immunity (Raaby Gammelgaard et al., 2021[93]). Although previous studies have highlighted the significant antitumor activity of the cGAS-STING pathway (Lv et al., 2024[70]), recent findings show that a triple therapy combining IFN-γ, STING agonist, and anti-PD-1 antibody significantly enhances cGAS/STING expression, IFN-β secretion, and tumor cell apoptosis. This combination therapy also synergistically activates multiple immune cell populations to boost antitumor immunity. These discoveries provide a novel strategy for GC immunotherapy and expand the therapeutic potential of IFN-γ-based tumor immunotherapy (Hosseinzadeh et al., 2024[45]).

#### Augmenting CD8+ T cell infiltration and cytotoxic activity in HCC

HCC poses considerable health risks (Zhang et al., 2024[137]). Tachykinins (substance P and NKA) and their receptors (NK1R/NK2R) are expressed not only in the nervous system but also in tumors (Nizam and Erin, 2018[82]). IFN-γ enhances T cell responses by upregulating NKA/NK2R expression in dendritic cells via STAT1 (Kitamura et al., 2012[55]). Recent findings indicates that IFN-γ enhances NK2R expression in CD8+ T cells via STAT1 signaling, while the absence of NK2R compromises their ability to combat tumors (Shen et al., 2023[104]). These results reveal the IFN-γ-STAT1-NK2R axis as a potential antitumor pathway.

#### Remodeling the TME of colorectal cancer and enhancing ICB sensitivity

Researchers have developed an engineered probiotic strain to enable targeted and prolonged IFN-γ release, preserving its immunostimulatory functions within malignant tissues (Li et al., 2024[60]). The team implemented a Synchronized Lysis Circuit (SLIC), which has a genomically integrated system enabling population-regulated IFN-γ delivery (SLIC-IFN-γ) (Savage et al., 2023[97], Vincent et al., 2023[117]). Research findings indicate that a single intratumoral administration of SLIC-IFN-γ enables targeted IFN-γ release in colorectal cancer models. When combined with bacterial adjuvants, this approach effectively stimulates myeloid cells, within both the TME and draining lymph nodes. Notably, IFN-γ monotherapy delivered via bacterial vectors exhibits potent antitumor activity in MHC-I-deficient and IFN-γ signaling-deficient models by activating NK cells. Furthermore, SLIC-IFN-γ enhances CD8+ T cell expansion in distant tumors and increases the sensitivity of advanced cancers to PD-1 blockade. No significant adverse effects have been observed. The combined strategy of PD-1 blockade with engineered bacterial-mediated IFN-γ delivery presents a promising avenue for cancer immunotherapy (Li et al., 2024[60]).

#### Combined chemotherapy enhances the killing effect on neuroblastoma

Neuroblastoma is responsible for roughly 10% of cancer-related fatalities in pediatric patients (Smith et al., 2010[107]). Combining chemotherapy with immunomodulators agents to leverage the innate immune system and potentially imporve therapeutic responses has remained a central focus of research in this field (Zeki et al., 2023[135]). Recent investigations have found the localized IFN-γ delivery to tumor sites via silk biomaterials can effectively inhibit neuroblastoma growth. Notably, combining IFN-γ with vincristine exhibits synergistic tumoricidal effects. These findings not only validate IFN-γ antitumor mechanisms but also provide novel insights for developing combination immunotherapy strategies against neuroblastoma (Zeki et al., 2023[135]) (Table 1(Tab. 1); References in Table 1: Castro et al., 2025[15]; Chaudhari et al., 2024[17]; Homann et al., 2022[44]; Hosseinzadeh et al., 2024[45]; Jia et al., 2021[48]; Li et al., 2024[60]; Shen et al., 2023[104]; Zeki et al., 2023[135]; Zhu et al., 2024[138]).

### Attenuating pro-tumorigenic effects 

Although IFN-γ exhibits significant potential in tumor immunotherapy, its potential tumor-promoting effects (Song et al., 2019[108]) still require focused attention and mitigation. Recent studies demonstrate that combination therapies can effectively attenuate IFN-γ-mediated protumorigenic effects, thereby improving both treatment safety and efficacy. These findings provide crucial evidence supporting the clinical translation of IFN-γ-based therapies (Xie et al., 2023[126]).

#### Counteracting IFN-γ-driven ICI resistance in liver cancer therapy

Studies indicate that NSDHL links cholesterol metabolism to tumor progression, with context-dependent pro- or anti-tumor effects (Gabitova-Cornell et al., 2020[32]). Regorafenib, a tyrosine kinase inhibitor, has been authorized for the treatment of hepatocellular carcinoma (HCC) in patients with sorafenib resistance (Gordan et al., 2024[40]). In HCC models, regorafenib-ICI combination enhances CD8+ T cell infiltration (Shigeta et al., 2020[105]). Recent studies indicate IFN-γ induced by ICIs suppresses NSDHL expression, which results in SREBP1 activation and elevated TGF-β1 production. This process diminishes T cell cytotoxicity while facilitating Treg infiltration, ultimately contributing to ICI resistance. Regorafenib counteracts this by modulating the IFN-γ/NSDHL/SREBP1/TGF-β1 axis, restoring ICI efficacy in HCC (Xie et al., 2023[126]), representing a novel strategy for overcoming IFN-γ-mediated immunosuppression in cancer therapy.

#### Inhibiting IFN-γ-mediated PD-L1 expression in multiple cancers

Traditional Chinese Medicine has been utilized worldwide for centuries in cancer treatment, with natural compounds-especially botanical extracts-continuing to serve as vital resources for innovative medical discoveries. Atractylodes macrocephala (AM), or "Baizhu" in Chinese, refers to the dried rhizome of AM Koidz, a perennial plant first recorded in the ancient text “Shennong Ben Cao Jing” over two millennia ago. This herb harbors a variety of bioactive substances (Li et al., 2022[63]), including the sesquiterpene lactones atractylenolide- II (AT-II) (Deng et al., 2021[24]). Experimental studies indicate that the combination of AT-II and IFN-γ therapy enhances the colorectal cancer immune-microenvironment by blocking IFN-γ-driven activation of the NF-κB p65/PD-L1 signaling pathway, effectively suppressing tumor progression and lung metastases (Wu et al., 2024[125]). This combination not only offers a novel immunotherapeutic approach but also provides a strategic solution for overcoming IFN-γ-mediated immunosuppressive resistance.

Triptolide, a natural compound, exhibits potent antitumor activity in cancer (Wu et al., 2024[124]). Using FDA-approved Pluronic F127 hydrogel, researchers co-delivered triptolide with IFN-γ. In triple-negative breast cancer, this combination showed deep tumor penetration, where triptolide blocked IFN-γ-induced PD-L1 upregulation, suppressing tumor growth (Cai et al., 2023[14]). The synergy enhanced antitumor CD8+ T cell responses, demonstrating a novel approach to modulate the dual effects of IFN-γ.

IFN-γ has demonstrated considerable therapeutic potential in cancer therapy, driving its clinical evaluation across diverse cancers. However, inconsistent trial results highlight the need to optimize its application for different tumor types (Table 2(Tab. 2); References in Table 2: Alberts et al., 2008[3]; Artis and Spits, 2015[6]; Gautam et al., 2022[36]; Reinisch et al., 2002[94]; Schmeler et al., 2009[99]; Vahdat et al., 2007[115]; Zarogoulidis et al., 2013[134]; Zibelman et al., 2023[139]).

## Notes

Yi Zhang and Li Yang (Biotherapy Center and Cancer Center, The First Affiliated Hospital of Zhengzhou University, Zhengzhou, China; E-mail: fccyangl1@zzu.edu.cn) contributed equally as corresponding author.

## Declaration

### Consent for publication

Not applicable.

### Competing interests 

No potential conflicts of interest are disclosed.

### Funding 

This work was supported by grants from the National Natural Science Foundation of China (grant numbers 82350121, 82573155), Science and Technology Innovation Team Support Plan from Henan Province (grant number 25IRTSTHN039), Young and middle-aged Health Science and Technology Innovation Talents in Henan Province (grant number LJRC2024012), Outstanding Young Talents Project from Henan Province (grant number 2523004210210), Top Talent Plan from Zhengzhou University, and Funding for Scientific Research and Innovation Team of The First Affiliated Hospital of Zhengzhou University (grant number ZYCXTD2023013).

### Authors' contributions

Conceived and revised the review: Li Yang, Yi Zhang

Wrote the paper: Jiahui Cui 

### Declaration of Generative AI and AI-as sisted technologies in the writing process 

During the preparation of this work the author(s) used DeepSeek to correct the grammatical and typographical errors in the manuscript. 

## Figures and Tables

**Table 1:**
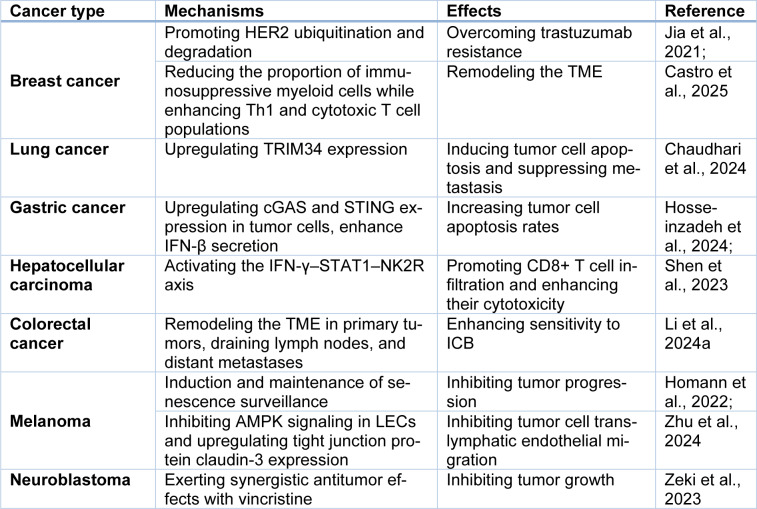
IFN-γ exerts antitumor effects through distinct mechanisms across various tumor types

**Table 2:**
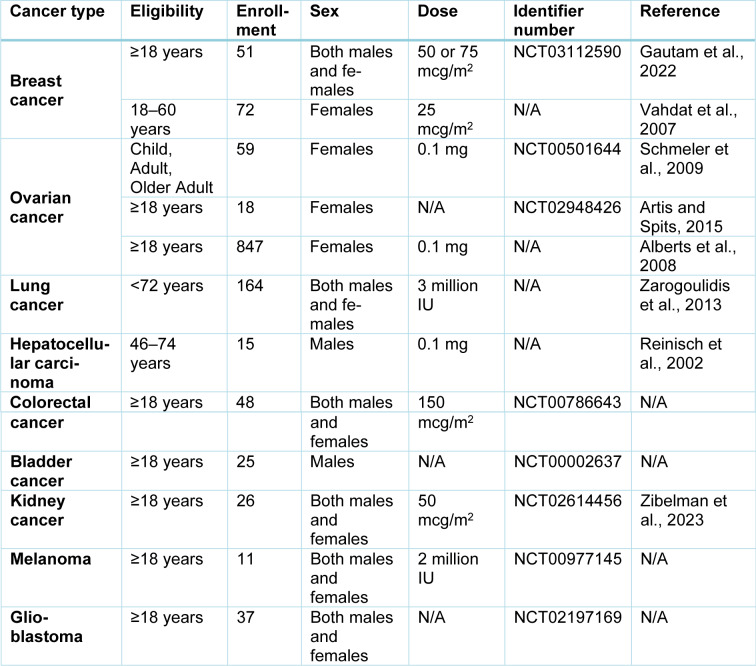
Clinical trials on IFN-γ for cancer treatment

**Figure 1:**
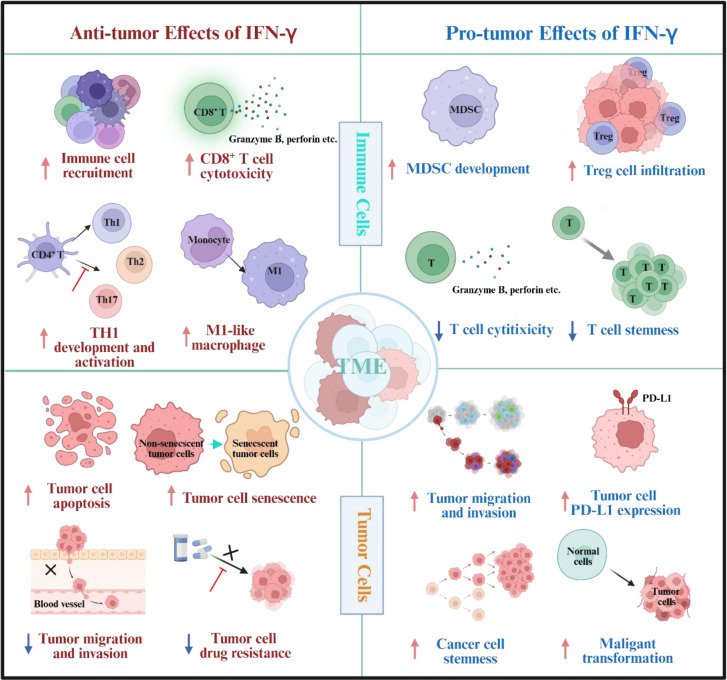
Graphical abstract
